# Inhibition of miR-142-5P ameliorates disease in mouse models of experimental colitis

**DOI:** 10.1371/journal.pone.0185097

**Published:** 2017-10-23

**Authors:** Nicolette W. Duijvis, Perry D. Moerland, Cindy Kunne, Monique M. W. Slaman, Faas H. van Dooren, Esther W. Vogels, Wouter J. de Jonge, Sybren L. Meijer, Kees Fluiter, Anje A. te Velde

**Affiliations:** 1 Tytgat Institute for Liver and Intestinal Research, Academic Medical Center (AMC), Amsterdam, the Netherlands; 2 Bioinformatics Laboratory, Department of Clinical Epidemiology, Biostatistics and Bioinformatics, Academic Medical Center, Amsterdam, the Netherlands; 3 Department of Pathology, Academic Medical Center (AMC), Amsterdam, the Netherlands; 4 Department of Clinical Genetics, Academic Medical Center (AMC), Amsterdam, the Netherlands; University of South Carolina School of Medicine, UNITED STATES

## Abstract

**Background:**

MicroRNAs (miRNAs) are epigenetically involved in regulating gene expression. They may be of importance in the pathogenesis of inflammatory bowel disease (IBD). The aim of this study was to determine the role of miRNAs by their specific blocking in the CD4+CB45RB^hi^ T-cell transfer model of chronic experimental colitis.

**Methods:**

Colitis caused by transfer of WT CD4+CD45RB^hi^ T cells in severe combined immunodeficiency (SCID) mice shares many features with human IBD. Colonic miRNA expression levels were measured at three time points in colitic mice, where a time-dependent upregulation of multiple miRNAs was seen. To inhibit these miRNAs, specific locked-nucleic-acid-modified (LNA) oligonucleotides were administered in further experiments at the moment the mice demonstrated the first signs of colitis. As controls, PBS and a scrambled sequence of anti-miRNA were used. Genome-wide expression analyses were also performed in order to detect candidate target genes of miR-142-5p, of which inhibition resulted in most effective amelioration of colitis.

**Results:**

Anti-miR-142-5p reduced colitis and related wasting disease when administered in the T-cell transfer model, reflected in reduced weight loss and a lower disease activity index (DAI). In further validation experiments we also observed a higher survival rate and less colonic histological inflammation in the antagomir-treated mice. Moreover, by genome-wide expression analyses, we found downstream activation of the anti-inflammatory IL10RA pathway, including three genes also found in the top-20 candidate target genes of miR-142-5p.

**Conclusion:**

In conclusion, CD4+CD45RB^hi^-transfer colitis induces miR-142-5p. Blocking miR-142-5p reduced colitis and prevented wasting disease, possibly by activation of the IL10RA pathway.

## Introduction

Inflammatory bowel diseases (IBD) are chronic relapsing inflammatory disorders of the intestine, and affect an estimated 1 million residents in the USA, and 2,5 million in Europe. There are several mouse models of experimental colitis that can be used to study IBD.[1, 2] In this study, the CD45RB transfer colitis model was chosen because it closely reflects the altered pattern of gene expression observed in IBD.[3]

MicroRNAs (miRNAs) play a role in the epigenetic regulation of gene expression. These are short non-coding RNA molecules with a length of 18–25 nucleotides, acting as posttranscriptional regulators. They repress gene expression by interacting with the 3’ untranslated region of specific mRNAs. Some miRNAs are widely expressed whereas others are more specific to a subset of cells.[4, 5] Over 60% of all protein-coding genes are controlled by miRNAs.[6] Guo et al.[7] demonstrated that changes in mRNA levels closely reflect the impact of miRNAs on gene expression and indicate that destabilization of target mRNAs is the predominant reason for reduced protein output. MiRNAs are known to only cause minor adjustments in expression of their direct target genes.[8] Despite these limited direct effects, minor changes in expression can lead to more pronounced gene expression variations further downstream.[9]

It is known that miRNAs play a critical role in immunity.[10–12] In IBD, miRNA expression differs between ileal and colonic CD.[13] An overview by Pekow and Kwon[14] gives a summary of studies that examine miRNAs in IBD. As expected, general inflammation-associated miRNAs are differentially expressed in active disease, such as miR-146a (involved in innate Toll-like receptor (TLR) responses[15]), miR-192 (highly expressed in colonic tissues with active UC[16]), and miR-21 (upregulated in intestinal tissues of IBD patients[17]).

Several studies demonstrated that miRNAs can be efficiently silenced. Krützfeldt *et al*[18] have showed that intravenous administration of blocking locked nucleic acid (LNA) oligonucleotides (antagomirs) against miR-16, miR-122 and miR-194 resulted in marked reduction of corresponding microRNA levels in several different organs of mice. Silencing miRNAs by this method seemed to be specific, efficient and long lasting. Targeting miRNAs as a therapeutic intervention could be a beneficial new treatment.

In the current study, we identify miRNAs differentially expressed during the development of chronic colitis in a mouse T-cell transfer model. Furthermore, we investigate the effect on colitis after blocking the expression of selected miRNAs. By studying tissue mRNA expression after miRNA silencing, both in vivo target genes and pathways targeted by miR-142-5p have been determined.

## Materials and methods

### Animals

All experiments were approved by the animal-welfare committee of the Academic Medical Center (AMC), Amsterdam, the Netherlands. Experiments were performed using 8-week old female SCID mice (BALB/c background) and male RAG1 knockout mice (C56bl/6 background), as indicated in the results section. Both these mice strains lack T- and B-cells and are thus compromised in their adaptive immune responses. When these mice receive CD4^+^CD45RB^hi^ T cells, devoid of regulatory cells that are found in CD4^+^CD45RB^low^ T cell population, they develop colitis. In our model we adhered to the protocol of Read and Powrie.[19]. In brief, homogenized spleen cells of BALB/c or C56bl/6 mice were depleted by negative selection, using an antibody mix of anti-mouse B220, CD8, CD11b and anti-rat-IgG-coated magnetic beads (Dynal, Life technologies Europe BV, Bleiswijk, the Netherlands). CD4^+^ cells were then positively isolated. The CD4^+^ T cells were sorted into a CB45RB^hi^ and CD45RB^lo^ population with a FACS ARIA (BD Biosciences, Breda, the Netherlands), by staining for anti-mouse CD45RB FITC and CD4 PerCP (BD Biosciences). CD4^+^CD45RB^hi^ cells were washed and 4*10^5^ cells were injected in SCID or RAG1-/- recipient mice. In a control group, 2*10^5^ CD4^+^CD45RB^low^ T cells were injected together with 4*10^5^ of CD4^+^CD45RB^hi^ T cells. All animals were kept in randomly located individually ventilated cages (IVC) on a controlled 12-hour light/dark regimen in the animal facility of the AMC. Mice were randomly distributed over cages with a maximum of five mice per cage. All mice were fed a standard rodent chow and acidified water ad libitum. For euthanasia, we adhered to the standard protocols used in the animal facility of the AMC: anesthesia was performed by administering 2 parts water, 1 part hyponorm and 1 part midazolam i.p., 0,07ml per 10 grams of mouse body weight, before sacrificing the mice by means of cervical dislocation. During the experiments analgesics were not used as these could possibly affect the outcomes of the study.

### Compounds

In order to block miRNAs, antagomirs were created (sequences in Table 1). Antagomirs are locked nucleic acid (LNA) and 2’-O-Methyl-RNA mixmer oligonucleotides with full phosphorothioate backbones[20] (Ribotask ApS, Odense, Denmark). These antagomirs have a defined sequence to block a specific miRNA. A scrambled miRNA, predicted not to bind to any mRNA targets, was generated and used as a miRNA compound control, while mice that haven’t received any CD4+CD45RB cells and thus did not develop colitis were used as a disease model control. Antagomirs were administered intraperitonally, 5mg/kg, dissolved in 0.9% saline.

**Table 1:** Locked nucleic acid (LNA) oligonucleotide sequences.

Compound	Sequence (5’-3’)	Purity
Scrambled LNA	5’-C a u G u g C u g C u a A c a A u u T a c C	>95%
Anti-miR-203	5’-C u a G u g G u c C u a A a c A u u T c a C	>95%
Anti-miR-146B	5’-A g c C u a T g g A a u T c a G u u C u c A	>95%
Anti-miR-142-5p	5’-A g u A g u G c u T u c T a c T u u A u G	>95%
Anti-miR-223	5’-T g g G g u A u u T g s C a a A c u G a c A	>95%

The four most significantly upregulated miRNAs in transfer colitic mice versus transfer non-colitic mice were selected, and anti-miRNA LNA sequences and a scrambled LNA sequence were created for use in further experiments. LNA moieties are capitalized, while lowercase bases are 2’OMeRNA.

### Experiments

Several experiments with different outcome measures were performed (Table 2). In the first experiment, 14 mice were injected with only CD4^+^CD45RB^hi^ cells to induce colitis, and 14 control mice with CD4^+^CD45RB^hi^ plus CD4^+^CD45RB^low^ cells. The mice were sacrificed at three different time points, when colitic mice have lost around 5%, 10% and 25% of their body weight compared to control mice. From both groups and at each time point, the three most representative mice (based on body weight and colon histology) were chosen for further mRNA and miRNA microarray experiments. Based on these results, antagomirs were created against the top-4 upregulated miRNAs in this colitis-transfer model (miR-142-5p, miR-146b, miR-203, and miR-223; experiment #2). In order to study the role of these miRNAs in the progression of colitis, antagomirs were administered twice a week to groups of five transfer-colitic mice at first signs of disease (5% body weight loss; +/- 3 weeks after T-cell transfer). Body weight and survival were checked and animals were sacrificed when they reached the humane endpoint, defined as over 15% body weight loss compared to starting weight, or otherwise after 9 weeks. One mouse in the scrambled and one mouse in the anti-miR-142-5p group had to be humanely euthanized before antagomir treatment, as they reached the humane endpoint. One mouse in the control group without colitis was excluded due to a non-disease and non-experiment related issue. In experiment #3, the antagomir showing the largest reduction in colitis, anti-miR-142-5p, was administered in male mice from a RAG1-/- background until death or sacrifice, using the scrambled antagomir as a control. This experiment validated that the results were not mouse-strain specific. In experiment #4 we aimed to study the effect in the colon on a transcriptional level of blocking miR-142-5p. The mice received antagomirs for five consecutive days starting when mice demonstrated a 10% reduction in their weight. The mice were sacrificed three days after the last injection.

**Table 2:** Experimental overview.

			Outcome					
Exp.	Mouse strain		Survival	Clin.	Hist.	mRNA expr.	miRNA expr.	Aims
1	SCID ♀	**Transfer-HI vs**. Transfer HI+LO		X	X	X	X	To compare intestinal mRNA and miRNA expression profiles between transfer-colitic (received CD4+CD45RB^hi^ T-cells) and transfer-non-colitic (received both CD4+CD45RB^HI+LO^ T-cells) mice, at three different time points during colitis development.
2	SCID ♀	**Transfer-HI vs**. no cell transfer vehicle/PBS scrambled anti-miR-203 anti-miR-142-5p anti-miR-146b anti-miR-223	X	X	X			To assess clinical impact of blocking selected miRNAs in transfer-colitic mice. Controls consisted of non-colitic mice (did not receive any T-cells), Phosphate buffered saline (PBS) administration, and a scrambled anti-miRNA control compound administration (predicted not to interact with any mRNA).
3	RAG1^-/-^ ♂	**Transfer-HI** scrambled vs. anti-miR-142-5p	X	X	X			To validate the clinical impact of blocking miR-142-5p in transfer-colitic mice from a different background and gender.
4	SCID ♀	**Transfer-HI** scrambled vs. anti-miR-142-5p				X		To assess colonic mRNA expression profiles following blocking of miR-142-5p in transfer-colitic mice.

Overview of the four transfer-colitis mouse experiments described in this study.

### Monitoring of colitis development

Mice with chronic transfer colitis develop a wasting disease. All mice were weighed three times a week, and progression of body weight loss was determined by percentage of weight loss from initial body weight. Animals were sacrificed at the indicated day in the experiment, or withdrawn from the study when they reached the humane endpoint. The disease activity index (DAI) ranges from 0 (no disease) to 4 (severe disease), and was assessed by averaging three separate scores, namely the severity of diarrhea, presence of blood in stool, and amount of weight loss (Table 3).[21] When colons were required for analyses, they were removed at sacrifice through a midline incision. The wet weight of the colon (corrected for mouse body weight) was used as a marker of disease-related intestinal wall thickening. A small part was fixed in formalin and embedded in paraffin for histology, the rest was snap-frozen for RNA isolation. Sections were stained with H&E for histological scoring by an experienced pathologist, blinded for treatment allocation. In the transfer model, inflammation was scored on a scale of 0–4, representing absent to severe inflammation.[19]

**Table 3:** DAI score.

Score	Diarrhoea	Blood	Weight loss
0	Normal stool	No blood	<1%
1	Soft stool		1–5%
2	Very soft stool	Blood in stool	5–10%
3	Fluid stool		10–15%
4	Empty, wet colon	Blood in stool and anal region	>15%

The DAI score is the average of three individual scores, combining severity of diarrhea (0 to 4 points), presence of blood in stool (0, 2 or 4 points) and percentage of body weight loss (0 to 4 points) into one score.

### MiRNA and mRNA microarray analysis

Total RNA was isolated using Trizol, according to manufacturer’s protocol (Trizol reagent, Invitrogen, USA). Quantity and quality of the RNA was checked by the Agilent 2100 Bioanalyzer platform (Agilent Technologies, Inc., Santa Clara, CA, U.S.A.). RNA integrity numbers (RIN) values of our samples ranged between 8.7 and 9.6. RNA concentration was measured using the Nanodrop ND-1000 spectrophotometer (Nanodrop Technologies, Wilmington, DE, U.S.A).

For the miRNA microarray experiment, samples were hybridized overnight onto miRXplore^™^ microarrays (Miltenyi Biotec GmbH, Bergisch Gladbach, Germany) at Miltenyi. Experimental and control samples (miRXplore universal reference, UR) were labeled with Hy5 and Hy3 respectively. Signal intensities were obtained using the ImaGene software (Biodiscovery) and normalized mean Hy5/Hy3 ratios were calculated using the PIQOR analyzer. The normalized Hy5/Hy3 ratios were log2-transformed. A systematic shift towards positive log-ratios on all arrays was corrected by fitting an equal variance two-component Gaussian mixture model (R package Mclust[22]) per array and subtracting the mean corresponding to the positive mode of the mixture model.

For the mRNA microarray experiment in experiment #1, additional quality control, RNA labeling, hybridization and data extraction were performed at Miltenyi Biotec GmbH (Bergisch Gladach, Germany). A total of 825ng of biotinylated cRNA was hybridized overnight onto the Agilent Whole Mouse Genome Oligo Microarray 4x44, using the Agilent Gene Expression Hybridization Kit (Agilent Technologies). Microarrays were scanned using an Agilent dual-laser DNA microarray scanner. The default Agilent normalization procedure, called Linear & Lowess, was applied. Normalized data were imported in Rosetta Resolver (Rosetta Biosoftware). An error-weighted 1-way ANOVA with Benjamini-Hochberg false-discovery rate multiple testing correction was used to identify differentially expressed genes between transfer-colitic and control mice at each of the three time points.

Amplification, labeling, hybridization and data extraction of material of experiment #4 were performed at ServiceXS (Leiden, The Netherlands) following the manufacturer's protocol. 1500ng of biotinylated cRNA was hybridized onto the Illumina MouseWG-6 v2 BeadChip (Illumina, Inc., San Diego, CA, U.S.A.). Hybridization and washing were performed according to the Illumina Manual “Direct Hybridization Assay Guide”. Scanning was performed on the Illumina iScan (Illumina, Inc., San Diego, CA, U.S.A.), while data extraction was performed with Illumina GenomeStudio v2011.1. Normexp-by-control background correction, quantile normalization, and log2 transformation[23] were performed on the Illumina sample and control probe profiles using the limma R package (version 3.10.2). The arrayQualityMetrics R package (version 3.10.0) was used to assess whether the microarray data were of good quality. Only probes detected (detection p-value < 0.05) on at least one array were included in the differential expression analysis. The illuminaMousev2.db R package (version 1.12.2) was used to update the probe annotation provided by Illumina.

For both the miRNA (#1) and the mRNA microarray experiment (#4), gene-wise linear models were fitted using the limma R package. Differential expression between transfer-colitic and control mice at each of the three time points and between anti-miR-142-5p and scrambled anti-miR treated mice, respectively, was assessed via an empirical Bayes moderated t-test. Ingenuity Pathway Analysis (IPA Spring Release 2016, QIAGEN) was used to identify upstream transcriptional regulators.

### Statistical analysis

All data are given as means ± SEM unless indicated otherwise. A non-parametric two-tailed Mann-Whitney U test was used to analyze differences between groups. Kaplan-Meier curves were compared using a log-rank test. A p-value <0.05 was considered statistically significant. The statistical analysis was performed using Graphpad Prism V5.01.

## Results

### MiRNAs are differentially expressed during CD4+CD45RB^hi^ transfer colitis

**Fig 1:**
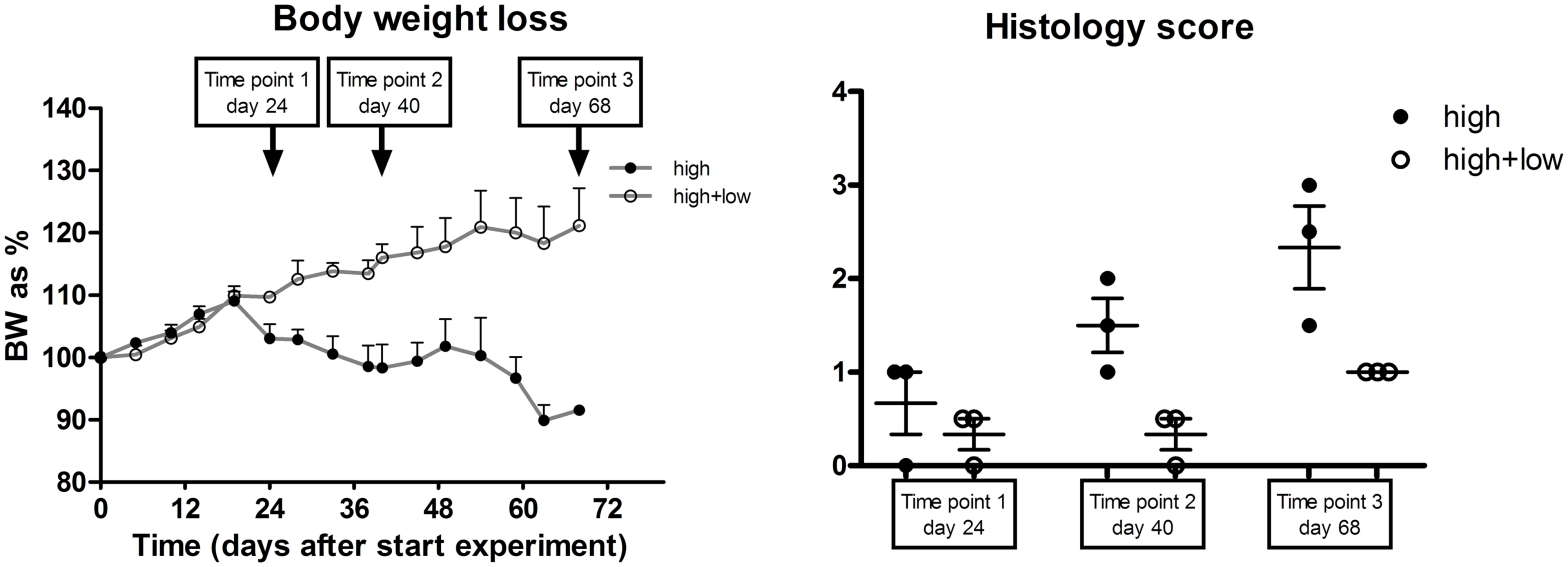
MiRNA expression in transfer-colitic mice. (A) Body weight loss over time in colitic (CD4+CD45RB^hi^) and non-colitic (CD4+CD45RB^hi+lo^) mice, after transfer of cells. Time point 1 marks a 5% difference in body weight between groups, while time point 2 and 3 mark 10% and 25% difference, respectively. Colitic mice start losing weight about three weeks after transfer of cells, while non-colitic mice keep gaining in weight. (B) Histological score of mice at the three different time points. Intestinal histological inflammation increases over time in colitic mice.

**Fig 2:**
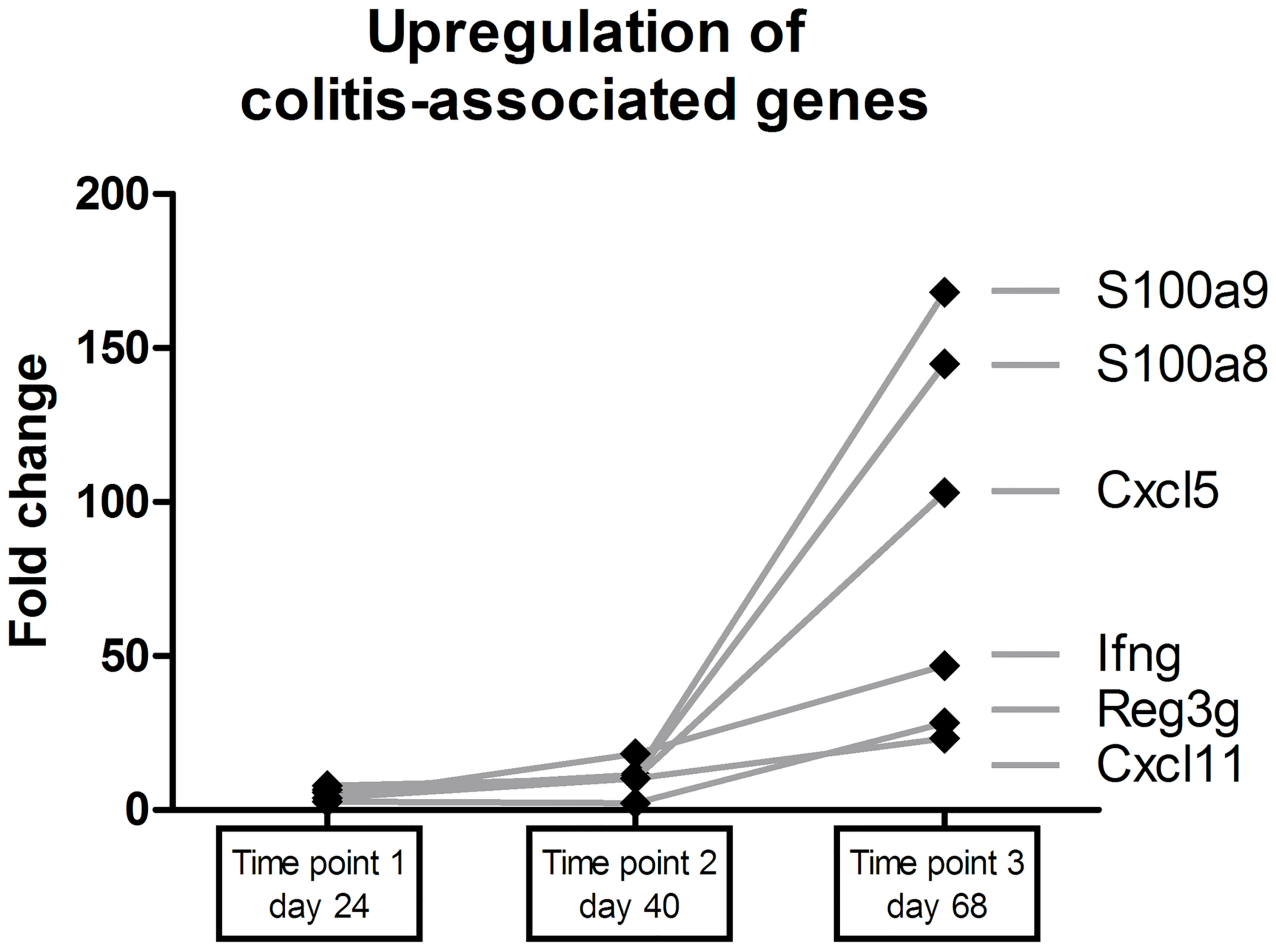
Upregulation of colitis-associated genes. Expression of colitis-associated genes increase following the increase in intestinal inflammation over time.

To determine miRNA expression during colitis development in transfer-colitic mice, 14 mice received CD4+CD45RB^hi^ cells to induce colitis while a control group of 14 mice received both CD4+CD45RB^hi^ and CD4+CD45RB^low^ cells (experiment #1), thus did not develop colitis. Fig 1A and 1B show the body weight loss of these mice at three established time points with related histology scores. Based on these parameters, the colons of the three most representative mice for each group were selected for further microarray analyses. At the mRNA level, a number of known transfer colitis-associated genes (such as S100A8, S100A9, Reg3g, CXCL5, CXCL11, and IFN-g[3]) were upregulated in colitic mice; this upregulation was related to the progression of the colitis (Fig 2). Next, the miRNA expression profiles were analyzed for all three time points. The top-11 differentially expressed (p<0.05) miRNAs between colitic and control mice at the third time point of sacrifice are listed in Table 4.

**Table 4:** Differentially expressed miRNAs in colon.

	Time point 1		Time point 2		Time point 3	
miRNA	FC	P-value	FC	P-value	FC	P-value
MIR-223	2.00	0.21	2.50	0.10	11.63	0.0003
MIR-142-5P	1.17	0.81	3.29	0.08	11.31	0.0019
MIR-146B-5P	2.60	0.16	2.36	0.20	10.70	0.0023
MIR-203	1.77	0.35	1.66	0.40	7.57	0.0041
MIR-18A	1.89	0.32	3.07	0.09	7.21	0.0064
MIR-7	1.14	0.82	2.19	0.18	5.43	0.0089
MIR-20A	1.54	0.44	1.54	0.44	4.92	0.010
MIR-142-3P	2.89	0.10	2.99	0.09	4.86	0.018
MIR-21	1.03	0.96	2.07	0.16	3.46	0.024
MIR-17	1.87	0.29	1.84	0.30	3.89	0.030
MIR-146A	1.67	0.46	4.26	0.05	4.41	0.046

Fold change (FC) of miRNA expression in colon of mice with CD45RB^hi^ transfer colitis versus non-colitic mice, at three different time points during colitis development. All 11 miRNAs with p<0.05 at time point 3 are listed.

### Amelioration of CD4+CD45RB^hi^ transfer colitis following antagomir treatment

**Fig 3:**
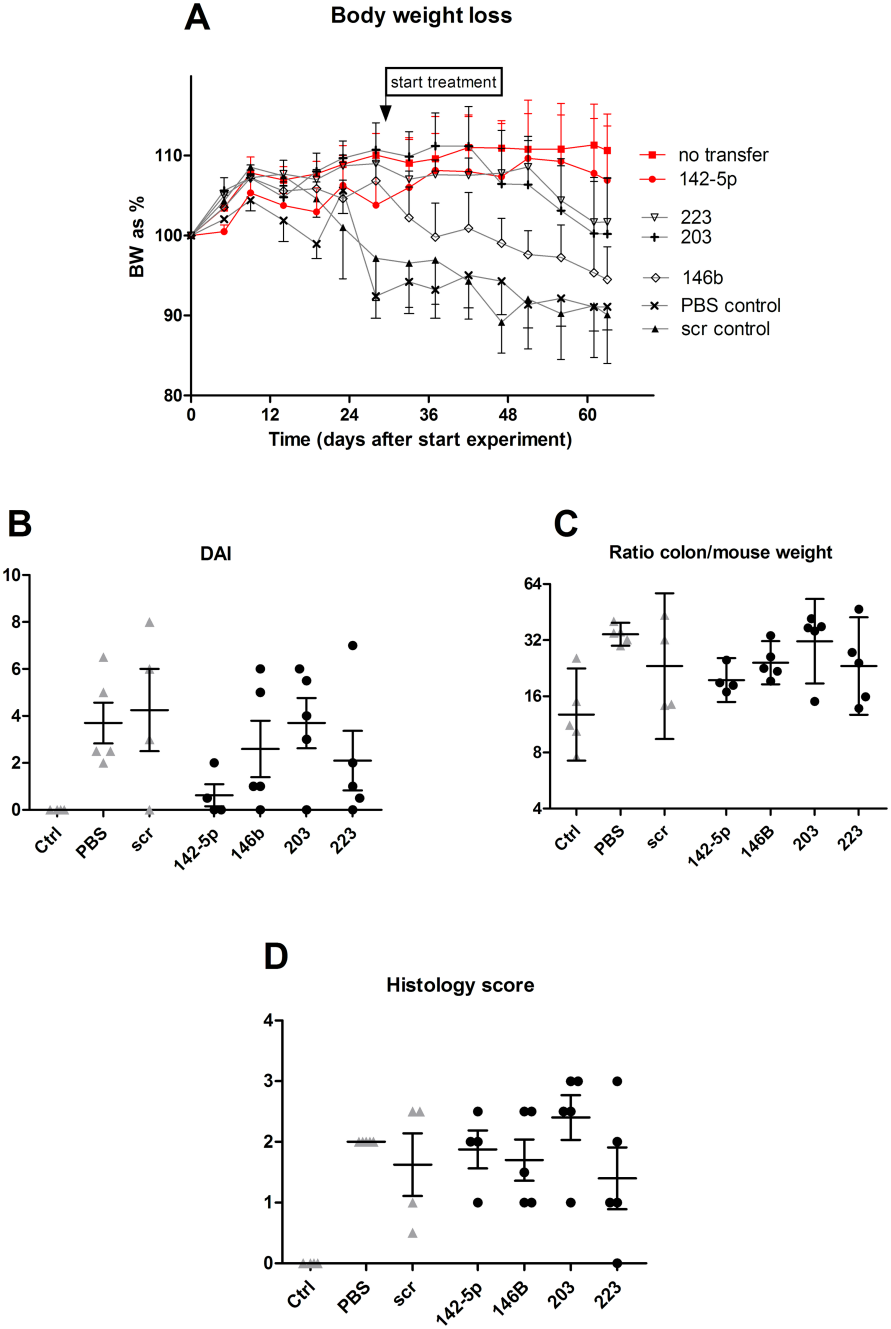
Disease parameters in antagomir-treated mice. Disease parameters in SCID mice treated with four different antagomirs, and control mice that have received either PBS or the scrambled LNA antagomir, or have received no cells at all (control, non-colitic mice). Despite that anti-miR-142-5p seemed to result in strongest amelioration of colitis, no significant differences were found when comparing antagomir-treated mice to scrambled LNA antagomir-treated mice. (A) Body weight loss over time. (B) Disease activity index. (C) Ratio of colon weight corrected for mouse weight. Geometric mean with 95% confidence interval. (D) Histology score.

**Fig 4:**
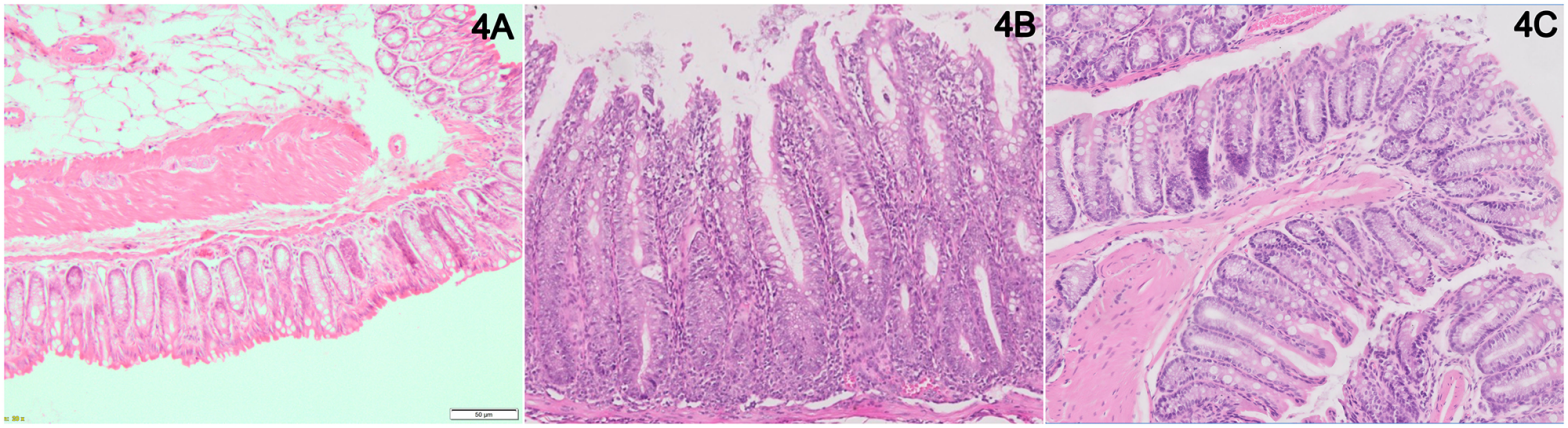
Representative HE-stained section of intestine of experimental mice. (A) Non-colitic mouse (B) Scrambled-treated mouse (C) Anti-miR-142-5p-treated mouse.

In order to establish specific effects of the four most upregulated miRNAs in our transfer colitis model, mice were treated with corresponding antagomirs directed against these miRNAs, twice a week after induction of colitis (at 5% body weight loss; experiment #2). A striking effect of the anti-miR-142-5p treatment was the healthy demeanor and appearance of these mice, as compared to colitic mice treated with other antagomirs. Animals receiving PBS or the scrambled LNA anti-miRNA showed most weight loss (Fig 3A), while the anti-miR-142-5p-treated and non-colitic mice showed least weight loss. The body weight curve of anti-miR-142-5p-treated mice is similar to the control mice that have not received any cell transfer, i.e. without induced colitis. Furthermore, when assessing the DAI, mice treated with anti-miR-142-5p exhibit hardly any active disease (Fig 3B). It is however important to note that the DAI consists of three separate scores: while the weight loss was reduced to the level of healthy mice, there was no difference in stool score between anti-miR-142-5p and other treatments (S1 Fig). No mice suffered from blood in stool, the third part of the DAI. Fig 3C and 3D show that there are no marked differences in colon weight and histology score between the colitis-induced groups, however when regarding individual sections of the intestine we do see that inflammation in anti-miR-142-5p-treated mice is less apparent in the entire colon compared to scrambled-treated mice. (Fig 4A–4C). By microscopically scoring all H&E-stained intestines of mice for granulocytes and mononuclear cells, we can estimate that, on a scale from 0 (no presence of cells) to 3 (extensive presence of cells), there were less granulocytes (1,75 in scrambled-treated mice, 1,25 in anti-miR-142-5p-treated mice) and less mononuclear cells (2,8 in scrambled-treated mice, 2,0 in anti-miR-142-5p-treated mice) present. We also see that these cells have not infiltrated as deeply into the different layers of the colon; while we see both granulocytes and mononuclear cells within the muscular layer and serosa of the scrambled-treated mice, these cells are limited to the mucosa, submucosa and in one case also the muscular layer of the intestine of anti-miR-142-5p-treated mice. In conclusion, mice treated with anti-miR-142-5p showed the least body weight loss, the lowest colon to body weight ratio and DAI score, and less cellular infiltration compared to mice treated with other antagomirs.

### Validation of treatment results of anti-miR-142-5p

**Fig 5:**
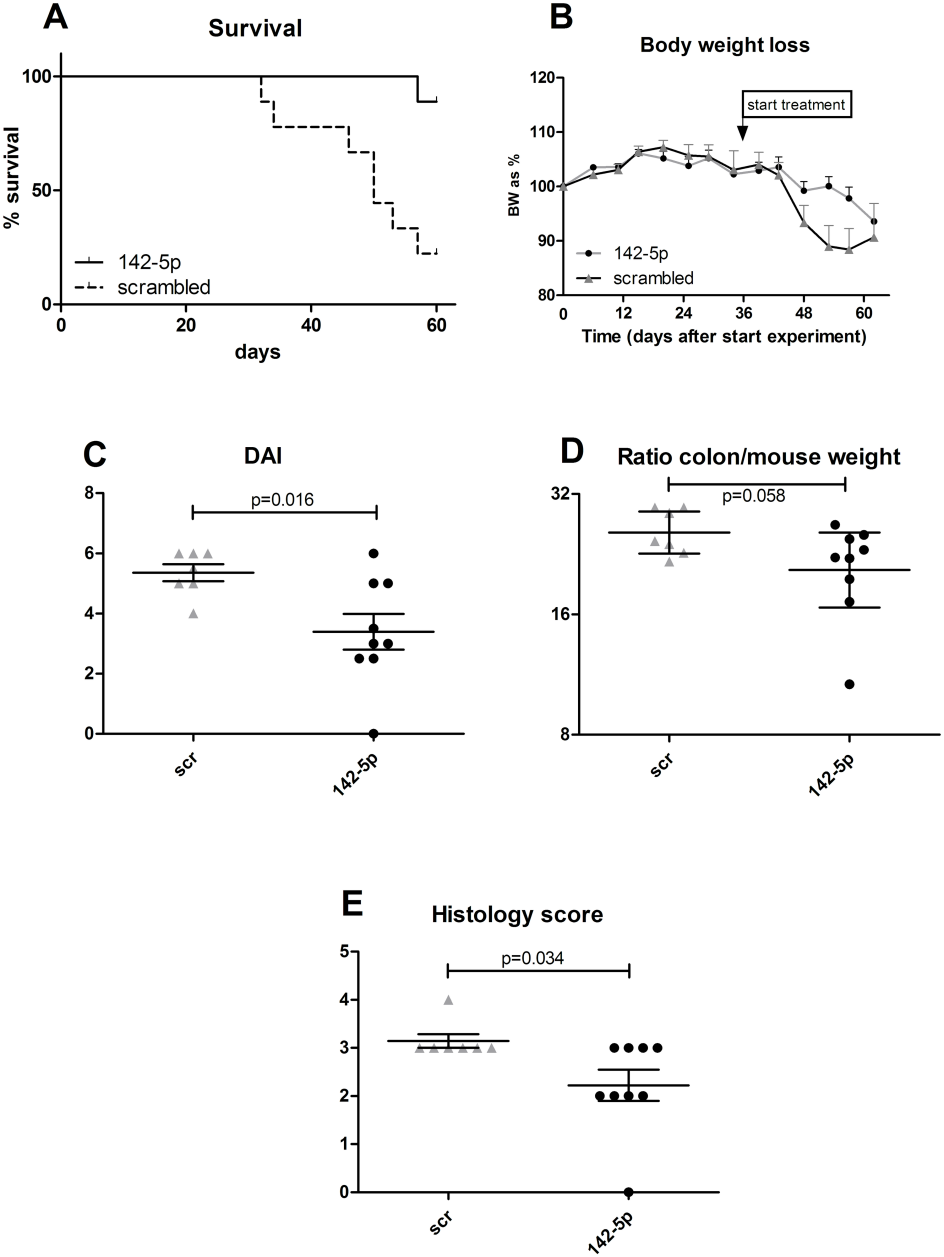
Disease parameters in anti-miR-142-5p-treated mice. Disease parameters in RAG1-/- mice treated with anti-miR-142-5p. (A) Survival of colitic mice treated with anti-miR-142-5p or the scrambled LNA-antagomir (control). Mice treated with anti-miR-142-5p show an improved survival. Log-Rank: P = 0.0026. (B) Body weight loss is less in anti-miR-142-5p-treated mice. (C) Disease activity index at death or sacrifice is significantly lower in anti-miR-142-5p-treated mice. (D) Ratio of colon weight corrected for mouse weight. Geometric mean with 95% confidence interval. (E) Histology score at death or sacrifice is significantly lower in anti-miR-142-5p-treated mice.

**Fig 6:**
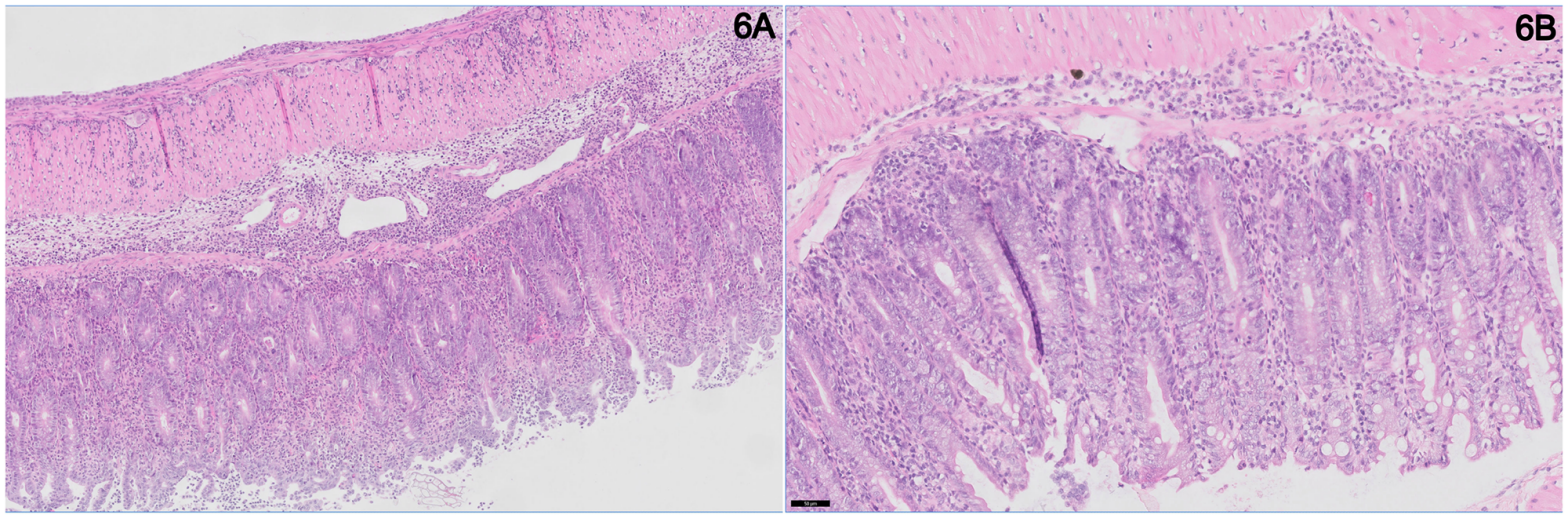
Representative HE-stained section of intestine of experimental mic. (A) Representative HE-stained section of intestine of scrambled-treated mouse. (B) Representative HE-stained section of intestine of anti-miR-142-5p-treated mouse.

To validate whether anti-miR-142-5p treatment shows similar effects in mice from a different background, we set up an experiment in which we treated ten male RAG1-/- mice with induced transfer colitis, and compared results to scrambled LNA treatment (experiment #3). In this experiment we also observed a clear effect of blocking miR-142-5p on the development of colitis as antagomir-treated mice demonstrated enhanced survival (Fig 5A) and less body weight loss (Fig 5B) compared to the scrambled-treated mice. The animals were sacrificed when they reached their humane endpoint (15% body weight loss compared to starting weight) or otherwise after 9 weeks. Again we clearly observed the healthy appearance of the anti-miR-142-5p-treated mice. When comparing the colon weight after sacrifice, we see a trend to lower colon-to-body-weight ratio in the anti-miR-142-5p-treated group (p = 0.058, Fig 5C). In addition, anti-miR-142-5p treatment lowered colonic inflammation (p = 0.034, Fig 5D) and resulted in a significantly lower DAI (p = 0.016, Fig 5E). Scrambled-treated mice showed apparent histological inflammation (Fig 6A), with crypt elongation, abscesses and immune cell infiltrates, while anti-miR-142-5p-treated mice display healthier intestinal mucosa (Fig 6B).

### Identifying candidate target genes of anti-miR-142-5p treatment

To find candidate target genes of miR-142-5p, we treated transfer-colitic SCID mice with anti-miR-142-5p or the scrambled anti-miR for 5 days daily, after establishing 5% weight loss (experiment #4). After mice were sacrificed at day 8, RNA was isolated from the colon and transcriptome profiling was performed. The top-20 most significantly upregulated and downregulated genes can be found in S1 and S2 Tables, respectively. Differentially expressed genes are likely to be either directly or indirectly regulated by miR-142-5p. Of the selected upregulated genes in S1 and S2 Tables, several genes are predicted to be directly regulated by miR-142-5p (targetscan.org): GDNF, CHIC1, BACE2, OCIAD2, PLAT, FAM162b, and MAN1A2.

**Fig 7:**
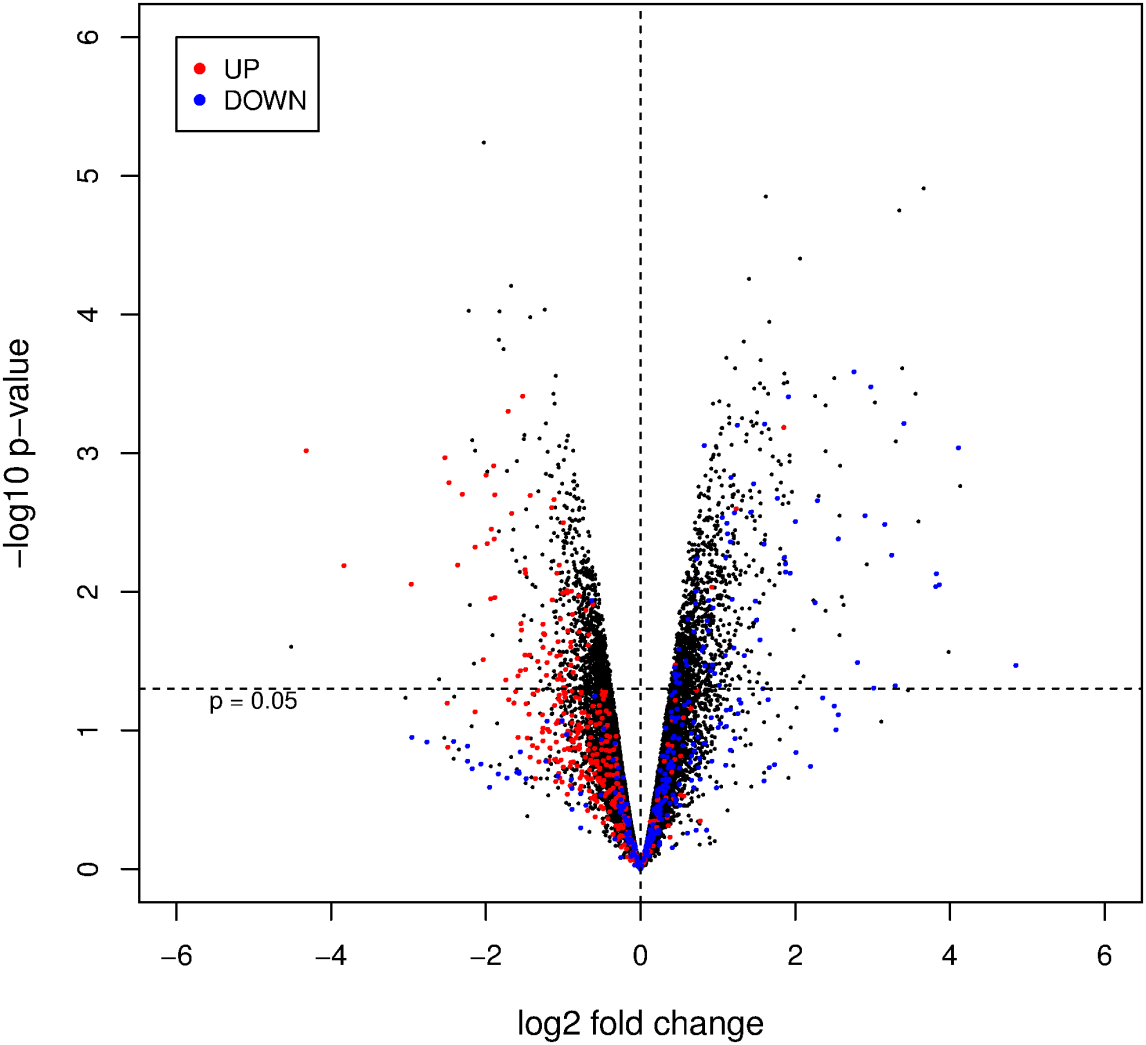
Volcano plot of genes affected by colitis and anti-miR-142-5p treatment. Volcano plot for the comparison of LNA anti-miR-142-5p treated mice versus scrambled LNA controls. Fold change is indicated on log_2_-scale on the x-axis, The nominal p-value is indicated on log_10_-scale on the y-axis. Genes that are regulated in the comparison of transfer-colitic (received CD4+CD45^hi^ cells) mice to non-colitic (received both CD4+CD45^hi^ and ^lo^ cells) mice (experiment #1, 3^rd^ time point).

Comparing the top-regulated genes of this experiment (in which treated and untreated colitic mice were profiled) with those of the mRNA microarray in experiment #1 (profiling colitic and non-colitic mice), we found a large overlap in the affected genes and an apparent reversed expression profile (Fig 7). A selection of genes, based on highest fold change after anti-miR-142-5p treatment with potential relation to inflammatory intestinal disorders or gut physiology, is shown in Table 5 (second and third column, respectively) and Fig 7. Of this table, Cyp2c55 and MAL (MyD88 adaptor-like) are the only genes predicted to be directly regulated by miR-142-5p (targetscan.org).

**Table 5:** Reversal of expression profile toward a healthy state after anti-miR-142-5p treatment.

Gene symbol	Fold change anti-miR-142-5p vs scrambled^a^	Fold change transfer colitic mice vs non-colitic mice^b^	Fold change transfer colitic mice vs non-colitic mice^c^
	UP		
Cyp2c55	28.8	-8.1	-4.8
MAL	17.5		-1.2
HMGCS2	17.2	-5.0	-1.5
Mettl7b	10.4	-2.4	
Sult1a1	8.9	-11.3	-10.0
Mep1a	7.9	-4.3	-2.7
Aldob (= Aldo2)	6.8	-9.0	-5.7
Aldh1a1	5.9	-2.2	-2.3
Hspa1a	5.8	-8.4	-19.5
Edn2	4.9	-3.1	-2.5
Sult1d1	4.8	-10.4	-3.6
Dpep1	4.2	-3.4	-4.8
	DOWN		
Sprr2d	-20.0	28.2	5.7
Serpinb11	-14.3	28.9	
S100a9	-7.8	168.1	225.4
Sprr2h	-5.8	29.5	14.5
Dlk1	-5.7	8.1	4.9
Sprr2b	-4.5	2.3	1.6

Selection of genes with potential relation to inflammatory intestinal disorders or gut physiology that are strongly up- or downregulated in colon mRNA analysis of anti-miR-142-5p-treated versus scrambled LNA treated colitic mice (2^nd^ column). A reversed expression profile is observed when comparing transfer colitic mice to control non-colitic mice (3^rd^ column). This reversed expression profile is also seen in data from our previously published study, also comparing transfer colitic mice to control non-colitic mice (4^th^ column).

Thus, mice treated with anti-miR-142-5p revealed a directional change towards a gene expression profile similar to non-colitic mice. An identical trend is observed when comparing with previously published data by our group, in which gene expression profiles were assessed in multiple mouse colitis models (Table 5, fourth column).[3]

In order to identify putative direct upstream regulators following induction of colitis (experiment #1) or after anti-miR-142-5p treatment in colitic mice (experiment #4), IPA Upstream Regulator Analysis was used. IPA predicts which upstream regulators are activated or inhibited to explain the upregulated and downregulated genes observed in the two transcriptomic datasets. For these analyses, we here selected the 250 most significantly affected genes based on adjusted P-value. When comparing mRNA expression profiles of transfer-colitic mice to non-colitic mice, the anti-inflammatory IL10RA is predicted to be inhibited as an upstream regulator (S3 Table). In all, 30 differentially regulated genes from our dataset fall in the IL10RA pathway, of which 28 have an expression direction consistent with inhibition of IL10RA. When comparing anti-miR-142-5p-treated to scrambled-treated transfer-colitic mice, we see an opposite relationship: IL10RA is predicted to be activated as an upstream regulator of genes affected by anti-miR-142-5p treatment. In this case, 15 out of 18 differentially regulated genes have an expression direction consistent with predicted activation of IL10RA. Among them are three genes that are found in the top-20 significantly upregulated genes after anti-miR-142-5p treatment (S1 Table): MEP1A, DPEP1 and ALDOB. are highlighted. Upregulated genes (log_2_(FC)>log_2_(3), adjusted p<10^−6^) are indicated in red, downregulated genes (log_2_(FC) < -log_2_(3), adjusted p<10^−6^) are indicated in blue.

## Discussion

### Upregulated miRNAs in disease

We investigated the expression profiles of miRNAs in a representative chronic murine colitis model for human IBD,[3] the CD4+CD45RB^hi^ T-cell transfer model. Several miRNAs were induced in the colon during colitis development, most significantly miR-223, miR-142-5p, miR-146B and miR-203. Inhibition of these candidate miRNAs showed that miR-142-5p is the most effective in reducing colitis, and could potentially be a new target in the treatment of IBD. The other miRNAs found to be upregulated in our experiments have already been described to be involved in several important (auto)-immune processes. MiR-223 is involved in granulocyte activation, and is overexpressed in naïve CD4+ T-lymphocytes in patients with rheumatoid arthritis.[24] MiR-146 is upregulated in monocytes in response to LPS and downregulates genes involved in the signal transduction pathway of TLR4 signaling,[15] and miR-203 is enhanced in skin of patients with psoriasis.[25]

In IL-10 knockout mice, another experimental animal model of Th1-mediated IBD, five out of the 11 miRNAs described in our current study (miR-223, miR-142-5p, miR-142-3p, miR-21 and miR-146a) were significantly elevated in severely inflamed colon.[26] MiR-223, miR-146a and miR-142-5p were among miRNAs expressed in colon biopsies and saliva shown to differentiate CD from UC.[27] In miR-142-deficient mice, this miRNA was identified as a specific regulator for CD4^+^ dendritic cell homeostasis and was demonstrated to cause a defect in priming of CD4^+^ T cells.[28]

### Silencing miR-142-5p

Silencing of miR-142-5p resulted in less weight reduction, a better survival and a reduced DAI score in our colitic mice, although histological inflammation seemed less pronounced in SCID than in RAG1-/- mice. Interesting to note is that, while inflammation has not fully disappeared in anti-miR-142-5p treated mice, the body weight curve is similar to that of healthy animals (Fig 3A). To learn more about possible pathways involved in this beneficial effect, we isolated RNA from the colon of untreated mice and mice treated with anti-miR-142-5p. Each miRNA has the potential to repress the expression of many different genes, and the observed phenotype of the mice receiving anti-miR-142-5p can be the result of interplay of multiple repressed targets. Genes that were differentially expressed upon miR-142-5p inhibition (Table 5) largely reflected a directional change towards a gene expression profile more similar to non-colitic mice (Table 5, Fig 7).

### Gene targets of miR-142-5p

Of the differentially expressed genes following anti-miR-142-5p treatment listed in Table 5, and S1 and S2 Tables, only a few were found to be known direct targets of miR-142-5p according to the TargetScan prediction database. Of these, MAL and GDNF can be linked to the pathophysiology of IBD: MAL (also known as TIRAP), acts as an adapter molecule between TLR4 and Myd88,[29] activating NF-kB which might play a critical role in the severity of disease. Recently, studies in Mal-deficient mice have demonstrated that these mice experience increased inflammation and severity in the DSS model of colon epithelial injury[30] and that they are more susceptible to oral infection.[31] It was therefore concluded that MAL is essential for the integrity of the intestinal barrier and maintenance of intestinal homeostasis. GDNF, glial-derived neurotrophic factor released by enteric glia cells, regulates apoptosis of enterocytes, and was found increased in inflamed IBD mucosa.[32]

Several other genes were also affected by anti-miR-142-5p treatment, meaning that they are possibly indirectly regulated by miR-142-5p. These affected genes are related to important pathways involved in metabolism, innate defense, intestinal homeostasis and epithelial differentiation. The gene that is most upregulated following anti-miR-142-5p treatment (Table 5) is Cyp2c55. Expression of Cyp2c55 was observed to be downregulated in certain intestinal cells in the spontaneous colitis of IL10RA knock-out mice.[33] Cyp2c55 is an enzyme that converts arachidonic acid (AA) to biologically active epoxyeicosatrienoic acids (EETs) that have potent anti-inflammatory properties.[34] Upregulation could thus result in an anti-inflammatory immune response in the gut and therefore, during colitis, blocking the miR-142-5p-mediated Cyp2c55 regulation might prove crucial to decreasing disease severity. Of the genes that were downregulated in this same experiment, two are members of the *SPRR2* family; these genes are involved in barrier function in different epithelia.[35] These SPRR2 genes are upregulated after inducing transfer colitis in SCID mice (Table 5, experiment 1).

### Upstream regulator IL10RA

By analyzing the top-250 differently expressed genes, we found that the most significant predicted upstream regulator that affects a similar gene set as anti-miR-142-5p treatment is IL10RA. IL10RA is one of the receptors for IL10, ligation of which results in an anti-inflammatory response known to play a role in IBD and other inflammatory diseases. While the CD4+CD45RO+^hi^ transfer colitis model showed a gene expression profile concordant with inhibition of IL10RA, treatment with anti-miR-142-5p was concordant with induction of IL10RA and reduction in colitis. This suggests that IL10RA is a key target of anti-miR-142-5p treatment and plays an important role in the improvement of intestinal inflammation.

Three of the genes directly regulated by IL10RA are in the top-20 genes upregulated upon anti-miR-142-5p treatment: MEP1A, ALDOP, and DPEP1. MEP1A (metalloproteinase meprin A) is a susceptibility gene for IBD, expressed in intestinal epithelium.[36] ALDOB (Aldolase B) is a member of the aldolase family, expression of which was found to be reduced in chronic transfer colitis.[37] DPEP1 (dipeptidase-1) has not been described to play a role in gastro-intestinal inflammation, but is expressed in colorectal cancer where it negatively correlates to aggressive disease and poor prognosis.[38]

### Wasting disease

Colitic mice treated with anti-miR-142-5p seemed healthy and groomed, unlike the general unhealthy appearance of mice treated with other compounds. The strongest disease marker influenced by anti-miR-142-5p treatment was body weight loss: even more so than the reduction in histological inflammation. We see that muscle wasting, or cachexia, is a serious problem in end-stages of severe diseases (e.g. cancer, infectious diseases (TBC/AIDS), or inflammatory disorders such as colitis).[39] Although the mechanism of cachexia is poorly understood, the immune system seems to play an important role. Op den Kamp et al. demonstrated that NF-κB activation leads to decreased skeletal muscle wasting and cachexia,[40] and both aforementioned genes MAL and GDNF affect NF-κB activation, that can lead to a cascade of effects in other pathways driven by NF-κB.[41, 42]. Other genes affected by therapeutic blocking of miR-142-5p as listed in S1 and S2 Tables that could also interfere with cross-talk interactions that NF-κB is a part of, are Nr5a2,[43] Cftr,[44] TNFRSF11b,[45] Adora1 (adenosine A1 receptor)[46] and PLAT.[47] In conclusion, in our model of blocking miR-142-5p in transfer-colitic mice, it is possible that systemic effects of the treatment are responsible for maintaining a healthy body weight, possibly by preventing immune-mediated cachexia.

## Conclusions

Taken together, in vivo blocking of miR-142-5p results in the up- or downregulation of candidate target genes in the colon. Treatment with anti-miR-142-5p prevented wasting disease and ameliorates disease severity of transfer-colitic mice, and modulated downstream targets of the IL10RA pathway, corresponding with IL10RA activation.

## Supporting information

S1 Fig Weight loss and stool score. Individual scores for weight loss and stool, per mice per treatment.
(TIF)

S1 Table Top 20 upregulated genes in anti-miR142-50 treated mice. Overview of most significantly upregulated genes in the colon after anti-miR142-5p treatment versus scrambled LNA treatment in CD45RB transfer colitic mice. Mice were injected i.p. for 5 consecutive days and sacrificed 3 days after the last injection. Resulting p-values were corrected for multiple testing using the Benjamini-Hochberg false discovery rate.
(DOCX)

S2 Table Top 20 downregulated genes in anti-miR142-5p treated mice. Overview of most significantly downregulated genes in the colon after anti-miR142-5p treatment versus scrambled LNA treatment in CD45RB transfer colitic mice. Mice were injected i.p. for 5 consecutive days and sacrificed 3 days after the last injection. Resulting p-values were corrected for multiple testing using the Benjamini-Hochberg false discovery rate.
(DOCX)

S3 Table Affected genes in IL10RA pathway. Affected genes in experiment #1 and experiment #4 that fall into the IL10RA pathway. In experiment #1, the anti-inflammatory IL10RAis predicted to be inhibited in colitic mice (IPA activation Z-score -4.600; p = 5.45E-28) while in experiment #4 anti-miR-142-5p treatment in colitic mice is predicted to result in activation of IL10RA (IPA activation Z-score 2.828, p = 4.45E-11).
(DOCX)
