# Supplementary figures and images for: Inhibition of miR-142-5P ameliorates disease in mouse models of experimental colitis
Source: PLoS One. 2017 Oct 23;12(10):e0185097. doi: 10.1371/journal.pone.0185097 (PMC5653202; doi:10.1371/journal.pone.0185097)

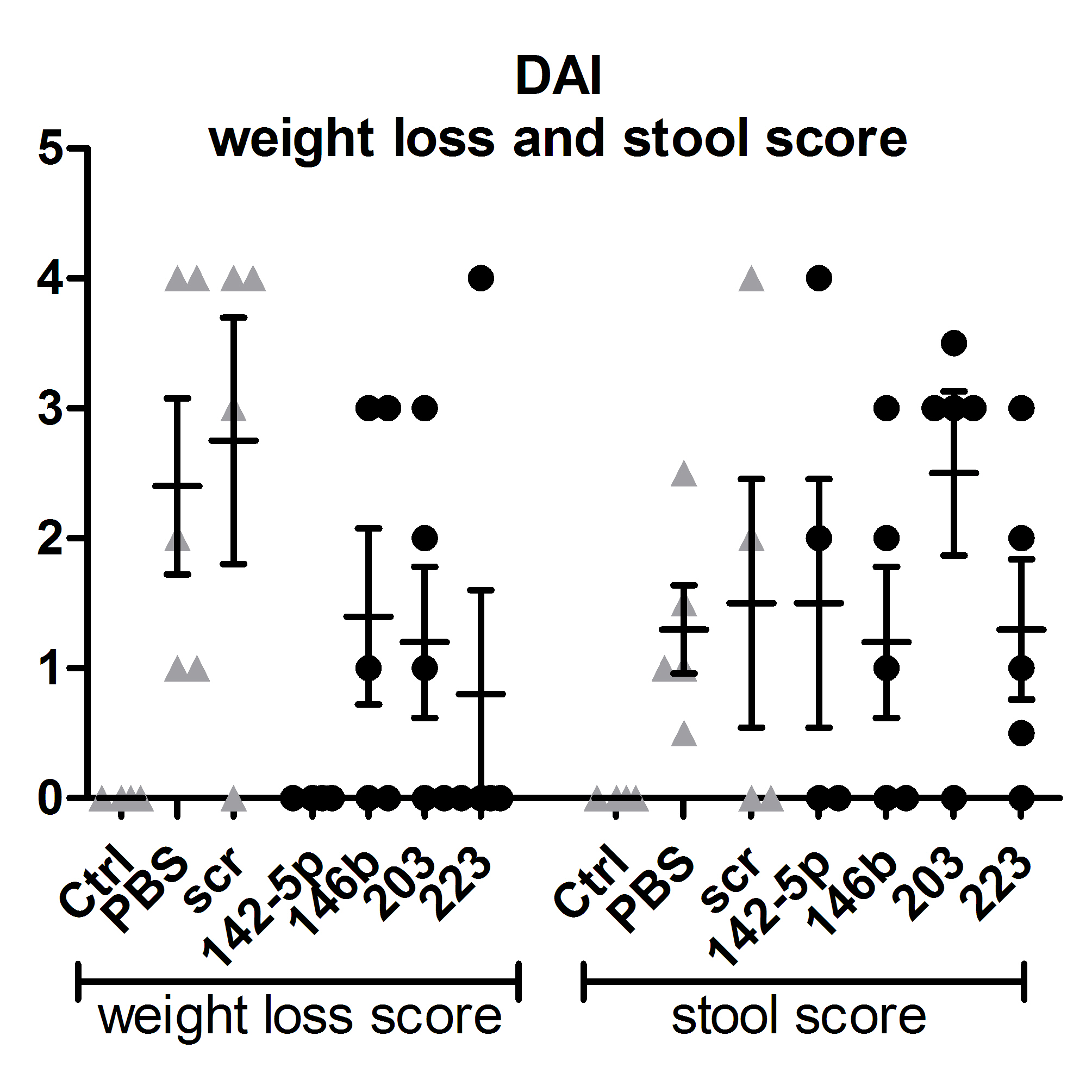

Supplement: S1 Fig — Individual scores for weight loss and stool, per mice per treatment. (TIF) [file pone.0185097.s001.tif]
