# Supplementary material for: Inhibition of miR-142-5P ameliorates disease in mouse models of experimental colitis
Source: PLoS One. 2017 Oct 23;12(10):e0185097. doi: 10.1371/journal.pone.0185097 (PMC5653202; doi:10.1371/journal.pone.0185097)
Supplement: S1 Table — Overview of most significantly upregulated genes in the colon after anti-miR142-5p treatment versus scrambled LNA treatment in CD45RB transfer colitic mice. Mice were injected i.p. for 5 consecutive days and sacrificed 3 days after the last injection. Resulting p-values were corrected for multiple testing using the Benjamini-Hochberg false discovery rate. (DOCX) [file pone.0185097.s002.docx]

Supplementary Table 1 – Top 20 upregulated genes in anti-miR142-5p treated mice

| **Illumina probe ID** | **Gene symbol** | **Fold change** | **P-value** | **Adjusted p-value** |
| --- | --- | --- | --- | --- |
| ILMN_1239800 | Gm1123 | 12.6 | 1.04E-05 | 0.089 |
| ILMN_2659994 | Gdnf | 3.1 | 1.35E-05 | 0.089 |
| ILMN_2754551 | Dpep1 | 4.2 | 3.53E-05 | 0.137 |
| ILMN_2816660 | Chic1 | 2.6 | 5.38E-05 | 0.168 |
| ILMN_2731444 | Hpd | 3.2 | 1.04E-04 | 0.187 |
| ILMN_2895312 | Eif4ebp3 | 2.5 | 1.50E-04 | 0.230 |
| ILMN_2720083 | Bace2 | 2.9 | 1.98E-04 | 0.230 |
| ILMN_2842843 | Amph | 2.2 | 2.06E-04 | 0.230 |
| ILMN_2707227 | Mettl7b | 10.4 | 2.14E-04 | 0.230 |
| ILMN_1224012 | Aldob | 6.8 | 2.29E-04 | 0.230 |
| ILMN_2746895 | O3far1 | 2.3 | 2.38E-04 | 0.230 |
| ILMN_2943722 | Ociad2 | 3.6 | 2.42E-04 | 0.230 |
| ILMN_1219796 | A4gnt | 5.7 | 2.57E-04 | 0.230 |
| ILMN_2505845 | Diras2 | 3.7 | 2.80E-04 | 0.230 |
| ILMN_2616479 | Mep1a | 7.9 | 2.94E-04 | 0.230 |
| ILMN_2634389 | Nr5a2 | 3.0 | 3.14E-04 | 0.230 |
| ILMN_2604310 | Eci3 | 2.8 | 3.21E-04 | 0.230 |
| ILMN_2705804 | Reg3b | 11.7 | 3.29E-04 | 0.230 |
| ILMN_1259577 | Cftr | 3.1 | 3.45E-04 | 0.230 |
| ILMN_1244458 | Tubal3 | 4.8 | 3.47E-04 | 0.230 |

**S1 Table.** **Top 20 upregulated genes in anti-miR142-5p treated mice**

Overview of most significantly upregulated genes in the colon after anti-miR142-5p treatment versus scrambled LNA treatment in CD45RB transfer colitic mice. Mice were injected i.p. for 5 consecutive days and sacrificed 3 days after the last injection. Resulting p-values were corrected for multiple testing using the Benjamini-Hochberg false discovery rate.
