# Supplementary material for: Inhibition of miR-142-5P ameliorates disease in mouse models of experimental colitis
Source: PLoS One. 2017 Oct 23;12(10):e0185097. doi: 10.1371/journal.pone.0185097 (PMC5653202; doi:10.1371/journal.pone.0185097)
Supplement: S2 Table — Overview of most significantly downregulated genes in the colon after anti-miR142-5p treatment versus scrambled LNA treatment in CD45RB transfer colitic mice. Mice were injected i.p. for 5 consecutive days and sacrificed 3 days after the last injection. Resulting p-values were corrected for multiple testing using the Benjamini-Hochberg false discovery rate. (DOCX) [file pone.0185097.s003.docx]

Supplementary Table 2 – Top 20 downregulated genes in anti-miR142-5p treated mice

| **Illumina probe ID** | **Gene symbol** | **Fold change** | **P-value** | **Adjusted p-value** |
| --- | --- | --- | --- | --- |
| ILMN_2619519 | Lce3b | -4.1 | 5.28E-06 | 0.089 |
| ILMN_1226398 | Fam162b | -3.2 | 5.77E-05 | 0.168 |
| ILMN_2976159 | Mreg | -4.7 | 8.37E-05 | 0.187 |
| ILMN_2513826 | Tnfrsf11b | -2.4 | 9.08E-05 | 0.187 |
| ILMN_3107114 | Stx3 | -3.4 | 1.62E-04 | 0.230 |
| ILMN_1236079 | Gm129 | -2.1 | 2.74E-04 | 0.230 |
| ILMN_2753750 | Lce3c | -2.9 | 3.61E-04 | 0.230 |
| ILMN_1254016 | Adora1 | -2.2 | 3.65E-04 | 0.230 |
| ILMN_2711772 | Micall2 | -2.2 | 4.32E-04 | 0.243 |
| ILMN_1241825 | Lor | -3.3 | 4.57E-04 | 0.243 |
| ILMN_2775937 | Plat | -2.8 | 6.86E-04 | 0.250 |
| ILMN_1248780 | Sprr2b | -4.5 | 7.32E-04 | 0.250 |
| ILMN_2959253 | 6030429G01Rik | -2.8 | 7.35E-04 | 0.250 |
| ILMN_2843029 | Man1a2 | -2.5 | 7.43E-04 | 0.250 |
| ILMN_1222803 | Hspa9 | -1.9 | 7.51E-04 | 0.250 |
| ILMN_2509674 | 1700030C10Rik | -1.9 | 8.15E-04 | 0.257 |
| ILMN_2626516 | Sprr2d | -20.0 | 8.54E-04 | 0.257 |
| ILMN_2604676 | Fam69b | -2.1 | 8.67E-04 | 0.257 |
| ILMN_2725781 | Abcg5 | -4.4 | 8.73E-04 | 0.257 |
| ILMN_3137291 | Lif | -2.0 | 9.11E-04 | 0.257 |

**S2 Table.** **Top 20 downregulated genes in anti-miR142-5p treated mice**

Overview of most significantly downregulated genes in the colon after anti-miR142-5p treatment versus scrambled LNA treatment in CD45RB transfer colitic mice. Mice were injected i.p. for 5 consecutive days and sacrificed 3 days after the last injection. Resulting p-values were corrected for multiple testing using the Benjamini-Hochberg false discovery rate.
