# Supplementary material for: Inhibition of miR-142-5P ameliorates disease in mouse models of experimental colitis
Source: PLoS One. 2017 Oct 23;12(10):e0185097. doi: 10.1371/journal.pone.0185097 (PMC5653202; doi:10.1371/journal.pone.0185097)
Supplement: S3 Table — Affected genes in experiment #1 and experiment #4 that fall into the IL10RA pathway. In experiment #1, the anti-inflammatory IL10RAis predicted to be inhibited in colitic mice (IPA activation Z-score -4.600; p = 5.45E-28) while in experiment #4 anti-miR-142-5p treatment in colitic mice is predicted to result in activation of IL10RA (IPA activation Z-score 2.828, p = 4.45E-11). (DOCX) [file pone.0185097.s004.docx]

Supplementary Table 3 – Affected genes in IL10RA pathway

| Experiment #1 transfer-colitic vs. non-colitic mice | | | Experiment #4 anti-miR-142-5p vs. scrambled | | |
| --- | --- | --- | --- | --- | --- |
| Genes in IL10RA pathway | Predicted direction of IL10RA | Exp. log ratio | Genes in IL10RA pathway | Predicted direction of IL10RA | Exp. log ratio |
| Ifng | Inhibited | 1.672 | Gm1123 | Activated | 3.659 |
| Il1a | Inhibited | 1.503 | Reg3a | Inhibited | 3.552 |
| Irg1 | Inhibited | 1.460 | Hao2 | Activated | 3.405 |
| Reg3g | Inhibited | 1.450 | Sult1a1 | Activated | 3.159 |
| F10 | Inhibited | 1.265 | Mep1A | Activated | 2.976 |
| Cxcl9 | Inhibited | 1.149 | Acer1 | Activated | 2.900 |
| Clec4e | Inhibited | 1.143 | Aldob | Activated | 2.763 |
| Lrg1 | Inhibited | 1.114 | Dpep1 | Activated | 2.058 |
| Cst7 | Inhibited | 1.097 | Hpgd | Activated | 1.954 |
| Iigp1 | Inhibited | 1.087 | Klk3 | Activated | 1.922 |
| Serpina3f | Inhibited | 1.056 | Reg3g | Inhibited | 1.853 |
| Lcn2 | Inhibited | 1.053 | Cftr | Activated | 1.645 |
| Clec5a | Inhibited | 0.889 | Nr5a2 | Activated | 1.597 |
| Il1b | Inhibited | 0.822 | Akr1c14 | Activated | 1.452 |
| Il6 | Inhibited | 0.804 | Art2b | Inhibited | 1.294 |
| Cd40 | Inhibited | 0.745 | Trpm2 | Activated | -1.117 |
| Nos2 | Inhibited | 0.732 | Tac1 | Activated | -1.929 |
| Tnf | Inhibited | 0.660 | Lcn2 | Activated | -1.992 |
| Fasl | Activated | 0.583 |  |  |  |
| Fas | Activated | 0.459 |  |  |  |
| Egf | Inhibited | -0.669 |  |  |  |
| Selenbp1 | Inhibited | -0.889 |  |  |  |
| Cyp2c55 | Inhibited | -0.910 |  |  |  |
| Pck1 | Inhibited | -0.911 |  |  |  |
| Aldob | Inhibited | -0.953 |  |  |  |
| Sult1a1 | Inhibited | -1.054 |  |  |  |
| Sycn | Inhibited | -1.057 |  |  |  |
| Cyp2c40 | Inhibited | -1.098 |  |  |  |
| Nov | Inhibited | -1.124 |  |  |  |
| Aadac | Inhibited | -1.152 |  |  |  |

**S3 Table.** **Affected genes in IL10RA pathway**

Affected genes in experiment #1 and experiment #4 that fall into the IL10RA pathway. In experiment #1, the anti-inflammatory IL10RA is predicted to be inhibited in colitic mice (IPA activation Z-score -4.600; p=5.45E-28), while in experiment #4 anti-miR-142-5p treatment in colitic mice is predicted to result in activation of IL10RA (IPA activation Z-score 2.828, p=4.45E-11).
